# Calcium or Sodium Carbonate Influence on Calcium Sulfoaluminate Clinker Hydration

**DOI:** 10.3390/molecules30132759

**Published:** 2025-06-26

**Authors:** Pilar Padilla-Encinas, Ana Fernández-Jiménez

**Affiliations:** Eduardo Torroja Institute for Construction Science, National Research Council (iETcc-CSIC), 28033 Madrid, Spain; maria.padilla@uam.es

**Keywords:** calcium sulfoaluminate clinker, CaCO_3_, Na_2_CO_3_, hydration

## Abstract

This work shows how the presence of calcium carbonate and sodium carbonate (5% and 20%) affects the hydration of a commercial calcium sulfoaluminate clinker (KCSA). For this study, water-hydrated pastes were prepared and the mechanical compressive strength and hydration rate were determined. The hydration products were characterised by XRD, DTA/TG, FTIR and SEM. The incorporation of CaCO_3_ can have a beneficial effect on the development of the mechanical strength of KCSA, especially at 90 days. It does not significantly alter the hydration kinetics and the hydration products formed are mainly ettringite and AH_3_. However, sodium carbonate has a detrimental effect, slowing down the hydration kinetics and reducing the development of mechanical strength, especially at early ages. The 20% Na_2_CO_3_ favours the formation of calcium aluminate, gaylusite and thenardite over ettringite. These phases are metastable in the presence of sodium and decompose to form calcite, alumina gel and a large amount of thenardite, which leaches out as efflorescence, causing microcracks and loss of strength in the material.

## 1. Introduction

The development of calcium sulfoaluminate (CSA) cements began in China in the 1970s. These cements consist of calcium sulfate clinker (KCSA ~85–75%) and calcium sulfate (preferably as gypsum ~16% to 25%) [1,2]. The main mineralogical phases of KCSA are ye’elimite (C_4_A_3_$\bar{s}$) and belite (C_2_S), with ferrite, calcium aluminate and calcium sulphate as secondary phases [3,4,5,6]. The hydration of CSA produces calcium monosulfoaluminate and aluminium hydroxide (microcrystalline) [6]. When KCSA is hydrated with excess water, ettringite and hydrated calcium aluminates (preferably katoite) are formed in addition to the above phases [3].

Many authors have studied the effect of calcium sulphate [7,8] and even sodium sulphate [9,10] on the hydration of CSA or KCSA. However, little is known about the effect of carbonates, both CaCO_3_ and Na_2_CO_3_.

In the case of Portland cement (PC), where the European standard EN 197-1 allows calcite to be added up to ≤5 wt% in CEM I and ≤35 wt% in CEM II, there are numerous published studies [11,12,13,14,15,16]. In the case of CaCO_3_ in Portland cement, it is known that a small fraction (~2% or 5%) can react with aluminate ions (C_3_A and C_2_(A,F) phases) present in Portland clinker to form calcium carboaluminate hydrates and promote ettringite stabilisation, which contributes to gap filling and strength development [11,12]. McDonald et al. [17] observed that the presence of more than 5% calcium carbonate in Portland cement can increase strength by 20% over the reference. The hydration products formed, scawtite and tilleyite, compete with each other and affect the strength of the product during hydration. High limestone additions (~20–35%) act as a diluent and have a more filler-like effect on PC hydration. Limestone is particularly effective as a surface for C-S-H nucleation [18]. Briki et al. [19] have shown that if the limestone is sufficiently fine, good strength performance can be achieved even with 20% replacement of clinker by limestone.

With regards to sodium carbonate (a highly soluble salt ~436 g/L), it has been shown that at low concentrations (below 2%) it acts as an accelerator of PC hydration, but at high doses (above 2%) it has a delaying effect [20]. It has been observed that Na_2_CO_3_ concentrations (2% and 4%) accelerate the hydration of alite and favour the precipitation of CaCO_3_ [21]. A study on the influence of Na_2_CO_3_ on the hydration of pure C_2_S and C_3_S phases [22] showed that the presence of sodium favours the precipitation of C-(N)-S-H gel. The 4% Na_2_CO_3_ accelerated the hydration and the development of initial strength in both silicates. Regarding the effect of these salts on aluminate phases, Sánchez-Herrero et al. [23] studied the effect of the incorporation of 4% Na_2_CO_3_ on the hydration of C_3_A. They observed that Na_2_CO_3_ favoured the formation of calcium monocarboaluminate (C_4_A$\bar{c}$H_11_) in the precipitation of hydrated cubic and hexagonal calcium aluminates.

Much less information and knowledge is available on the effect of both calcium and sodium carbonates on the hydration of CSA or KCSA.

In the hydration of CSA, it has been shown that CaCO_3_ in the form of calcite also influences hydration and the development of mechanical properties [24,25,26,27,28]. The addition of finely ground calcite generally reduces the setting time. Calcite promotes the formation of hemicarboaluminate and monocarboaluminate over monosulfoaluminate and the stabilisation of ettringite. In addition, calcite additions to CSA cement promote higher compressive strength at later ages compared to other quartz additions due to calcium carbonate reaction and porosity reduction [24].

Hargis et al. [28] investigated the effect of incorporating 10% calcium carbonate in the form of either calcite or vaterite into synthetic KCSA in the absence or presence of gypsum. The calcium carbonate reacted with monosulfoaluminate to form monocarboaluminate and ettringite. The reaction was about three times faster for vaterite than for calcite. This was related to the lower stability, higher solubility and higher surface area of vaterite. Vaterite and calcite showed significant reaction at 28 and 84 days respectively. Gypsum reduced the reactivity of both carbonates and promoted the early formation of ettringite at the expense of monosulfoaluminate, which limited the reactivity of calcium carbonate.

Less is known about the effect of Na_2_CO_3_ on the hydration of CSA or KCSA. Sánchez-Herrero et al. [29] observed that the presence of 4% Na_2_CO_3_ in the hydration of the pure ye’elimite phase reduced the development of mechanical strength and modified the hydration products, inhibiting the formation of AFt and favouring the formation of U phase. Other authors [9] indicate that the addition of 5% Na_2_CO_3_ to a CSA induced a false setting which, after remixing, delayed the setting times with respect to the reference. Efflorescence and thenardite precipitation were observed at long ages. There are also works analysing the behaviour of CSA exposed to the attack of a 10% Na_2_CO_3_ solution [30]. In these studies, it is observed that ettringite can react with sodium carbonate to form calcium carbonate, thenardite and aluminium hydroxide, causing cracking of the material and loss of strength.

In this work, the effect of these two carbonate salts (CaCO_3_ and Na_2_CO_3_) in low and high proportions (5% and 20%) in the hydration of an industrial clinker (without gypsum) has been studied in order to simplify the system and to elucidate the chemical reactions that take place. To this end, their effect on heat flow and total heat on the development of mechanical strength at short and long ages (3, 28 and 90 days) was studied, and the hydration products were characterised by XRD, FTIR, ATD/TG and SEM.

## 2. Results and Discussion

### 2.1. Isothermal Conduction Calorimetry

**Figure 1:**
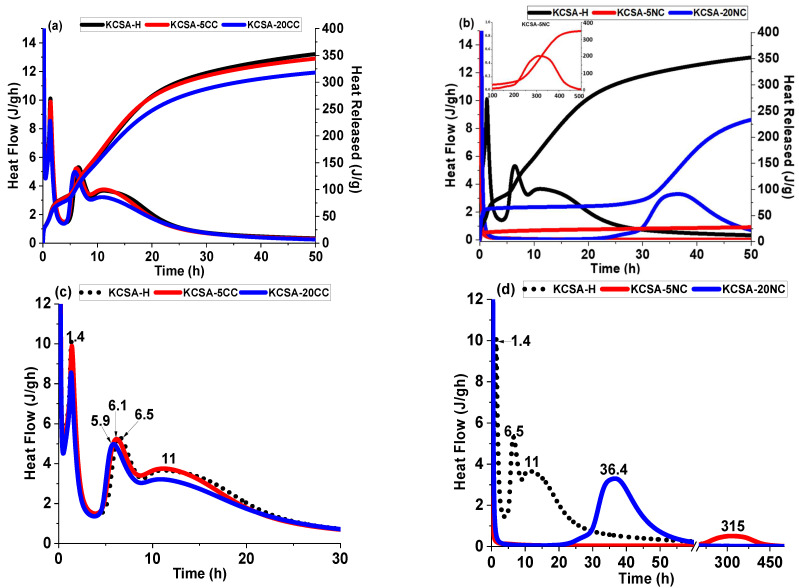
Heat evolution rate and total heat of pastes with 5% and 20% of different salts; (**a**) KCSA-CaCO_3_; (**b**) KCSA-Na_2_CO_3_; (**c**) heat flow extension KCSA-CaCO_3_; (**d**) heat flow extension KCSA-Na_2_CO_3_.

Figure 1 shows the heat flow and total heat curves obtained by isothermal conduction calorimetry. The reference paste (KCSA-H) shows the presence of several signals associated with KCSA hydration processes [4,5,31,32].

The heat flow curves of the CaCO_3_ incorporated pastes (Figure 1a,c) at both 5% and 20% are very similar to the reference, with three peaks and a shoulder detected. The first peak appears at 30 min, corresponding to the start of the hydration process, followed by a second peak appearing at 1.6 h for the KCSA-5CC paste and at 1.5 h for the KCSA-20CC paste. There is then an induction period (~2 h) followed by another peak with a maximum at 6.1 h for KCSA-5CC and at 5.9 h for KCSA-20CC. Finally, a shoulder is obtained for both pastes with a maximum at 11 h. The main difference between the 5% and 20% CaCO_3_ pastes is the higher intensity of the peaks of the KCSA-5CC paste.

After 50 h of testing, the heat of hydration of the pastes with calcium carbonate (KCSA-5CC= 344 J/g and KCSA-20CC= 317 J/g) is only slightly lower than that obtained in the absence of the salts (KCSA-H= 352 J/g, Figure 1a). These results indicate that calcium carbonate appears to have little effect on the initial hydration rate of KCSA, even at high levels of 20%. Calcium carbonate is a rather insoluble salt, so its initial reaction is very slow. The slight acceleration in obtaining the main peak with increasing amount of carbonate could be due to the fact that its presence provides additional nucleation sites for the different hydration products of KCSA, as indicated in previous studies by other authors [11,17,24,25,26,27,28,33,34].

The incorporation of Na_2_CO_3_ shows more relevant changes. The heat flow curves show the presence of two peaks (Figure 1b,d). The first peak at around 30 min is common to all pastes. This is followed by a long induction period for KCSA-5NC (~189 h), while for KCSA-20NC the induction period is 25 h. The second peak in the KCSA-5NC paste has its maximum at 315 h (≈13 days) and the KCSA-20NC paste has its maximum at 36.4 h.

This delay in the reaction is also observed in the heat curves (Figure 1b). At 50 h, the heat released by KCSA-5NC (28.8 J/g) and by KCSA-20NC (244 J/g) are much lower than those obtained by the reference sample or by the samples containing calcium carbonate. However, with increasing age, the heat values increase, reaching values of 250 J/g at 60 h for sample KCSA-20NC and 350.8 J/g at 500 h for sample KCSA-5NC. These heat increases correspond to the peaks observed in the heat flow curves and, as discussed later, based on the XRD results, are associated with the reaction of ye’elemite to give reaction products, type CAH_10_ in HCSA-20NC, and delayed ettringite formation in KCSA-5NC.

Sodium carbonate is a very soluble salt, so it is possible that when it dissolves it changes the pH of the medium. Further explanation of the possible reactions taking place in the KCSA-5NC and KCSA-20NC pastes will be discussed later. However, it should be noted that a similar behaviour in the heat flow curves was observed when KCSA was hydrated with NaOH solution at different concentrations [35,36].

### 2.2. Mechanical Strength

**Figure 2:**
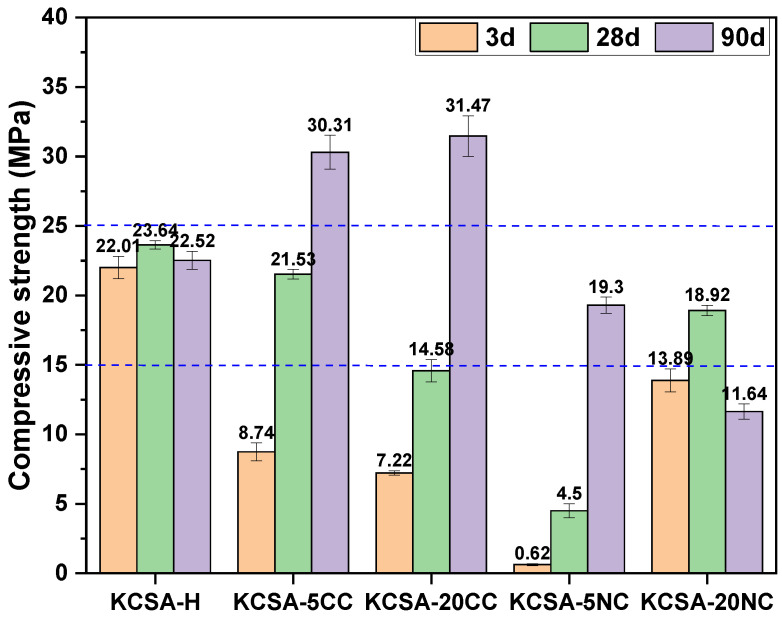
Mechanical compressive strength of paste.

Figure 2 shows the mechanical compressive strength values of clinker hydrated with water (reference) and with different concentrations of CaCO_3_ and Na_2_CO_3_ at the ages of 3, 28 and 90 days. The presence of CaCO_3_ leads to a decrease in strengths at 3 and 28 days compared to the reference, but these clearly increase at 90 days. In the case of sodium carbonate, the results obtained show, in general, lower mechanical strengths than the reference system at all ages studied; in fact, a drop in strengths is observed between 28 and 90 days for KCSA-20NC.

### 2.3. Paste Characterization

**Figure 3:**
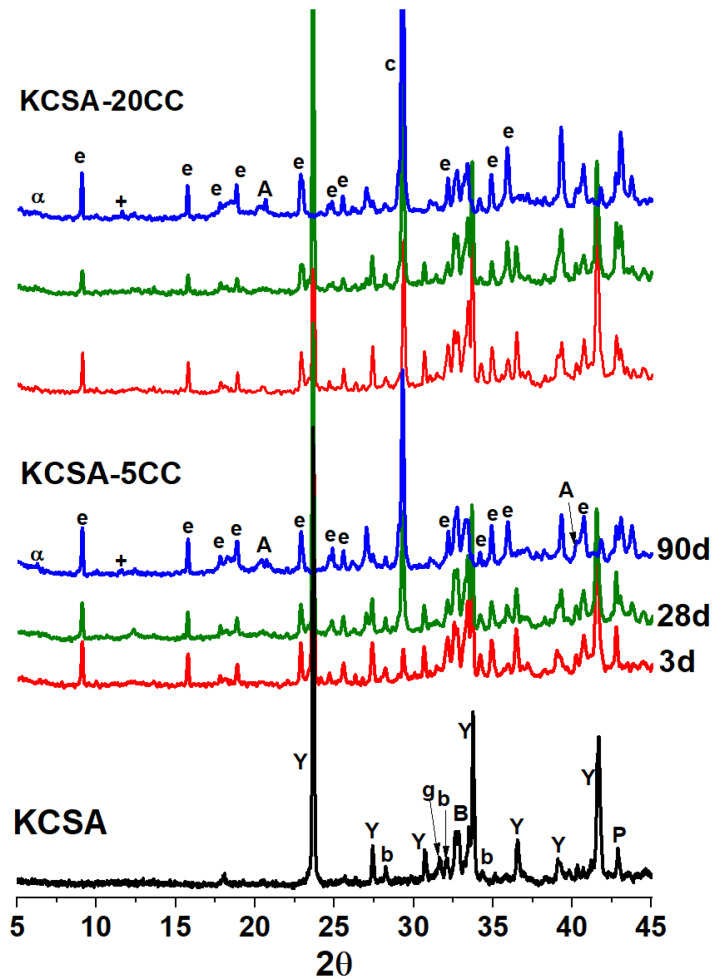
Diffractogram of KCSA-5CC and KCSA-20CC paste at 3, 28 and 90 days [Y: ye’elimite (PDF-33-0256); b: belite (PDF-86-0398); B: bredigite (PDF-36-399); g: gehlenite (PDF-35-755); P: periclase (PDF-4-829); e: ettringite (PDF-41-1451); A: gibbsite (PDF-74-1775); c: calcite (PDF- 86-2334); α: CAH_10_ (PDF-12-0408); +: gypsum (PDF-21-0816)].

**Figure 4:**
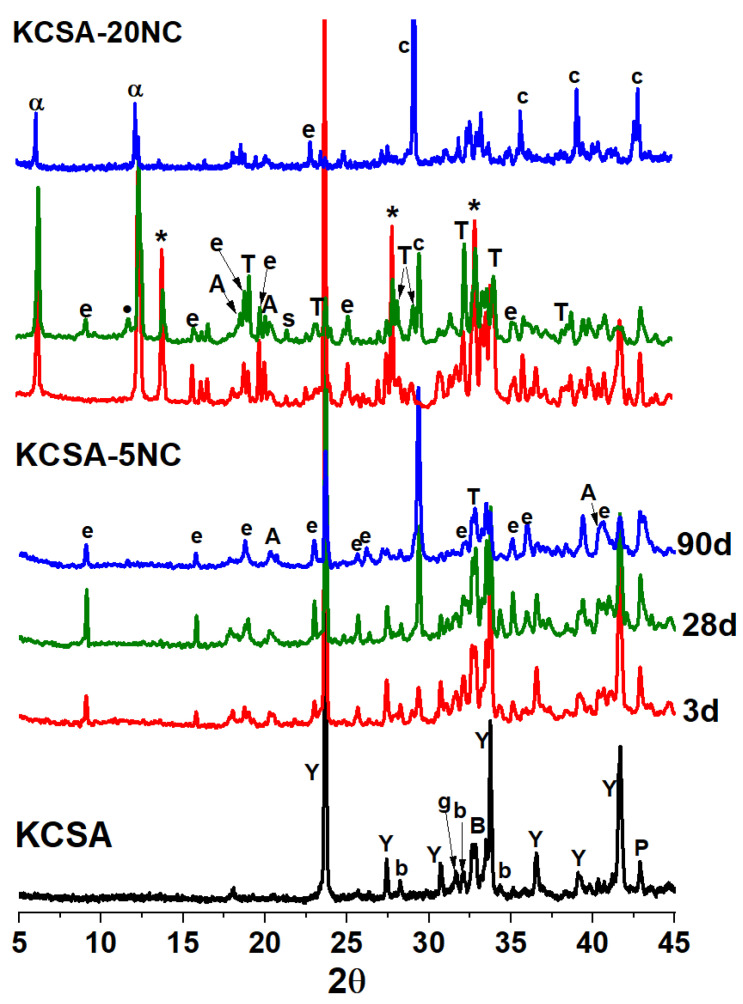
Diffractogram of KCSA-5NC and KCSA-20NC paste at 3, 28 and 90 days [Y: ye’elimite (PDF-33-0256); b: belite (PDF-86-0398); B: bredigite (PDF-36-399); g: gehlenite (PDF-35-755); P: periclase (PDF-4-829); e: ettringite (PDF-41-1451); A: gibbsite (PDF-74-1775); T: thenardite (PDF-37-1465); c: calcite (PDF- 86-2334); α: CAH_10_ (PDF-12-0408); *: gaylusite (PDF- 21-0343)].

Figure 3 and Figure 4 show the diffractograms corresponding to the clinker and the hydrated pastes in the presence of CaCO_3_ and Na_2_CO_3_ at 3, 28 and 90 days. The initial anhydrous KCSA shows ye’elimite (C_4_A_3_$\bar{s}$) as the main phase and belite (C_2_S), bredigite (Ca_7_Mg(SiO_4_)_4_), gehlenite (Al_2_Ca_2_O_7_Si), periclase (MgO) and traces of tricalcium aluminate (C_3_A) as minority phases.

The diffractograms of the pastes with the presence of calcium carbonate (KCSA-5CC and KCSA-20CC, Figure 3) show a decrease in the intensity of the peaks associated with gehlenite with increasing hydration time, reaching total consumption after 90 days. The peaks associated with the other anhydrous phases (belite, bredigite…) do not show significant changes in peak intensity. The presence of ettringite is observed as a reaction product whose peak intensity increases at 3, 28 and 90 days.

The presence of an aluminium hydroxide is also detected. The intensity and resolution of the AH_3_ peaks (2Ɵ values between 20–21°) are low, indicating a low degree of crystallinity [35] at all ages studied. The presence of these phases corresponds to the increase in strength over time.

At 90 days, a significant increase in the signal associated with calcite is also observed in both pastes (KCSA-5CC and KCSA-20CC, Figure 3), as well as the presence of small amounts of calcium aluminate hydrate and gypsum. The presence of calcium aluminate hydrate has been observed by other authors during the hydration of KCSA with water [32]. It should be remembered that KCSA contains not only ye’elimite but also other phases such as C_3_A, albeit in small amounts, which together with the available water influence the nature of the products formed [4]. The formation of these phases can densify the matrix and explain the significant increase in strength observed after 90 days (see Figure 2).

These results indicate that, from a mineralogical point of view, the addition of calcium carbonate does not participate significantly in the hydration reactions of KCSA, mainly due to its low solubility. The higher signal intensity of calcium carbonate after 90 days is associated with the formation of additional calcium carbonate. It is known that carbonation of ettringite can produce gypsum and AH_3_ in addition to calcite (phases detected in the 90-day diffractograms).

In the sodium carbonate pastes (Figure 4), the observed behaviour is somewhat different. In the KCSA-5NC pastes, the main reaction products are still ettringite and AH_3_. At 3 days, the intensity of the ye’elimite signal is very high, indicating a low degree of reaction. However, after 28 days the signal intensity decreases and that of ettringite increases.

This result confirms what was observed in the calorimetry, that the incorporation of 5% Na_2_CO_3_ delays the hydration reaction and the precipitation of ettringite. The delayed ettringite formation justifies the presence of the peak that appears at 13 days (315 h) in the heat flow curve (see Figure 1b,d). A similar behaviour was observed in previous work when KCSA was hydrated with a 1M NaOH solution [36]. At 90 days, the degree of reaction of the ye’elimite is higher, thus higher mechanical strengths are obtained. However, a slight decrease in the intensity of the ettringite signal is observed, associated with a carbonation process justified by the presence of calcite and thernardite.

In the KCSA-20NC pastes, after 3 days, the degree of reaction of the ye’elimite is low and the presence of calcium aluminate hydrate (CAH_10_) is detected as crystalline reaction products. The formation of this compound could be responsible for the peak at 36.4 h in the heat flow curves. The formation of a sodium calcium carbonate called gaylusite (Na_2_Ca(CO_3_) 2.5H_2_O) is also observed. This carbonate is metastable; it also appears at 28 days with less intensity, but it is no longer detected at 90 days.

Sodium carbonate is very soluble, when dissolved in the system (20% Na_2_CO_3_) the medium is saturated with C$O_{3}^{2-}$ and Na^+^ ions, which precipitate in the presence of calcium in the form of gaylusite. The presence of this phase has also been demonstrated in the hydration of PC at high alkali concentrations [37,38]. Its precipitation reduces the Na^+^ ion concentration in the medium, lowering the pH and creating conditions for the precipitation of ettringite (see KCSA-20NC diffractogram at 28 days). Gaylusite, as indicated, is a metastable phase and decomposes to form calcite. The water molecules released favour the further reaction of ye’elimite (almost 100% reacted after 90 days).

Free sulphates in the medium should form ettringite, but the amount of this salt formed is very low. Due to the high amount of sodium in the system, these sulphates precipitate as thenardite (as seen in the diffractogram at 28 days). At 90 days, calcite is the predominant mineral, and the amount of thenardite appears to have decreased because the thenardite has been released in the form of efflorescences. These surface efflorescences were removed by manual cleaning of the specimens prior to testing at 90 days or characterisation by the various techniques. The formation and leaching of this salt, together with the high degree of carbonation observed at 90 days, is attributed to the decrease in mechanical strength observed in this material between 28 and 90 days.

**Figure 5:**
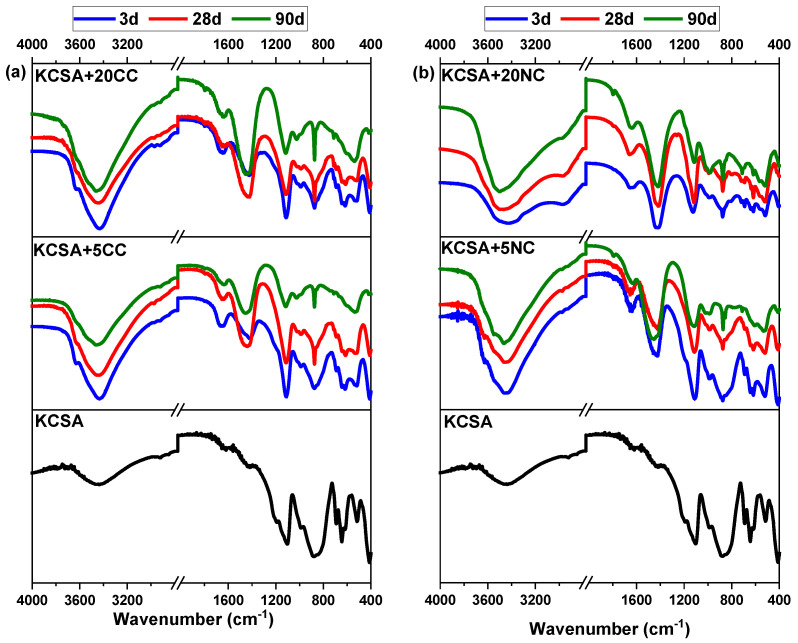
FTIR spectra of anhydrous KCSA and pastes substituted with different salts at 3, 28 and 90 days of hydration; (**a**) CaCO_3_ and (**b**) Na_2_CO_3_.

Figure 5 shows the FTIR spectra of KCSA samples hydrated for 3, 28 and 90 days with 5% and 20% CaCO_3_ and Na_2_CO_3_ salts.

The spectrum of anhydrous KCSA corresponds mainly to the bands associated with ye’elimite, with the vibrations of sulphate groups (S$O_{4}^{2-}$) at 1190, 1100 and 614 cm^−1^ and the bands at 990, 885, 820, 690, 645 and 413 cm^−1^ of aluminate groups (AlO_4_) [39,40]. Concerning the presence of C_2_S in KCSA, bands at 990, 845 and 514 cm^−1^ are observed due to the vibrations of Si-O groups [39]. The bands associated with the vibrations of the minority phase bonds overlap with the previous ones, making identification difficult.

The spectra of samples KCSA-5CC and KCSA-20CC are shown in Figure 5a. In general, the bands associated with the ye’elimite tend to decrease in intensity or shift slightly as a result of hydration, while the bands associated with the Si-O bonds show minor changes. The band at 1100 cm^−1^ indicates the presence of ettringite, which is confirmed by the presence of bands at 990, 885, 614 and 413 cm^−1^ and at high frequencies with bands at 3637 cm^−1^ related to OH stretching bonds, while peak at 1660 is related to OH bending bonds [41,42], and other ettringite bands overlap and are difficult to detect. AH_3_, (a phase difficult to detect by XRD due to its low degree of crystallinity) is characterised by a band at 3629 cm^−1^ associated with the vibrational frequency of the OH stretching bonds and a band at 1024 cm^−1^ corresponding to Al-O-H bending frequencies and bands at 614, 514 and 413 cm^−1^ related to AlO_4_ stretching. In addition, several signals corresponding to the C-O bond of the carbonates are formed from the above signals, obtaining bands at 1420 and 820 cm^−1^ [41,43,44,45,46].

In the case of the sodium carbonate pastes (Figure 5b), several signals are obtained corresponding to the different phases observed by XRD (ettringite, AH_3_, CAH_10_, thenardite and calcite). The signals at 3447 and 3266 cm^−1^ correspond to the OH bonds of ettringite, calcium aluminate and aluminium hydroxide. For ettringite different signals are obtained, at 1686 cm^−1^ the v_2_ symmetric vibrational band of water is formed, at 1100, 990 and 885 cm^−1^ the v_3_ S-O asymmetric vibrational band is observed and at 614 cm^−1^ the v_1_ Al-O symmetric vibrational band appears. Aluminium hydroxide shows several bands at different wavelengths. The band at 820 cm^−1^ corresponds to the asymmetric v_3_ Al-O vibration and at 690 and 614 cm^−1^ the symmetric v_1_ Al-O vibration is obtained. Calcium aluminate gives a signal at 1649 cm^−1^ corresponding to the v_2_ symmetric vibration of water, but due to the low intensity of these bands and their overlap it is difficult to make a clear assignment. Thenardite gives signals at 1100 cm^−1^ and 614 cm^−1^ corresponding to the v_3_ S-O asymmetric vibration and the v_4_ S-O deformation vibration respectively. Finally, the signal corresponding to calcite is obtained at 1478 cm^−1^ from the asymmetric v_3_ C-O vibration and at 885 cm^−1^ from the symmetric v_1_ C-O vibration [40,41,42,43,44].

**Figure 6:**
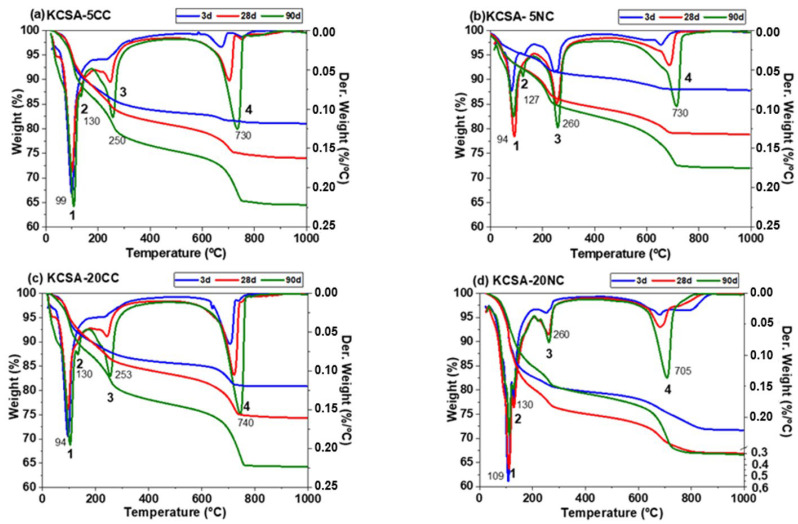
Differential thermogravimetric analysis (DTA) and thermogravimetric analysis (TG) of KCSA pastes substituted with different salts, (**a**) KCSA-5CC; (**b**) KCSA-5NC; (**c**) KVSA-20CC; (**d**) KCSA-20NC. Signals 1–4 have been labeled.

Figure 6 shows the results obtained by DTA/TG of KCSA pastes hydrated with 5% and 20% CaCO_3_ and Na_2_CO_3_ at 3, 28 and 90 days.

Mass losses up to ≈600 °C are generally related to water loss from the hydration products formed (ettringite, AH_3_, aluminates and hydrated calcium carboaluminates), while losses above 600 °C are mainly related to the release of CO_2_ from the carbonates present (calcite, sodium carbonate).

In multiphase systems, the dehydration temperatures of the individual phases often overlap [46,47]. Ettringite has a hexagonal prismatic structure with octahedral aluminium [Al(OH)_6_]^3−^ pillars bound by calcium and hydroxide ions ([Ca_6_Al_2_(OH)_12_]^6+^ and the sulphate and water molecules are located on the outer surface of the pillars ([3S$O_{4}^{2-}$·26H_2_O]^6−^). These water molecules are lost at around 100 °C. Water loss from AH_3_ results in a peak between 210–280 °C (usually centred at 250/270 °C). For CAH_10_, water loss is associated with a peak at 120 °C. Calcium monocarboaluminate (4CaO·Al_2_O_3_·CO_3_·11H_2_O) loses five water molecules between 60–200 °C (strong signal at 140 °C); the six water molecules in the octahedral layer are lost between 200–300 °C and CO_2_ at about 650 °C.

However, the positions of these peaks and their intensity can be slightly affected by the interactions of some phases with others, the conditions of the recording or the procedure for stopping the hydration reaction [47], making a good quantification difficult.

The DTA curves (Figure 6) show the presence of a series of signals corresponding to the decomposition of the different phases mentioned above and identified by XRD. The first signal (signal 1) at around 94–100 °C corresponds to the decomposition of ettringite. At around 130 °C, both calcium carbonate and sodium carbonate form a more intense signal (signal 2) at 90 days, associated with the decomposition of hydrated calcium aluminate (CAH_10_) [47]. Both signals overlap and it is difficult to determine the weight loss corresponding to each phase.

The signal appearing at temperatures close to 260 °C (signal 2) is associated with the decomposition of the aluminium hydroxide phase (AH_3_), a phase previously observed by XRD in all the pastes and at all the ages studied [47].

**Figure 7:**
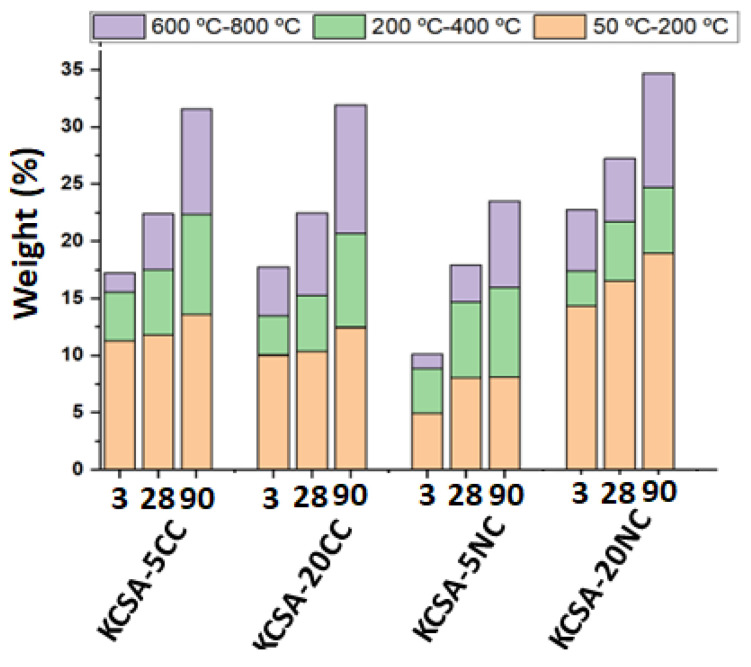
Results of percentage weight loss determined by TG.

In general, it is observed that the intensity of the signal associated with the decomposition of ettringite (signal 1) increases from 3 to 28 days and decreases slightly from 28 to 90 days, while the intensity of the signal of calcium aluminate increases at 90 days. This leads to an increase in weight loss between 50–200 °C with increasing hydration time, especially between 28 and 90 days (see Figure 7). Between 200–400 °C, an increase in weight loss is also observed with increasing hydration time, which is associated with the formation of a higher amount of AH_3_ over time. Finally, above 600 °C, a signal (signal 3) is obtained corresponding to the loss of CO_2_ from the carbonates [47]. It is observed in all pastes and its intensity increases with hydration time.

In terms of weight loss associated with carbonate formation (600–800 °C), a significant increase is observed in all cases between 28 and 90 days. These data coincide with the high calcite formation observed by XRD at 90 days.

Comparing the results in Figure 7 with the mechanical strength values (Figure 2), a good correlation is generally observed, with higher weight losses corresponding to higher strength values. For example, sample KCSA-5NC shows lower weight loss and lower strength; this behaviour is associated with a lower ye’elimite reaction as discussed above. However, KCSA-20NC shows the highest weight loss at all ages, which correlates well with the strength at 3 and 28 days, but not at 90 days. The drop in strength at 90 days is related to high carbonation and the formation of thenardite which leaches out as efflorescence in this sample.

**Figure 8:**
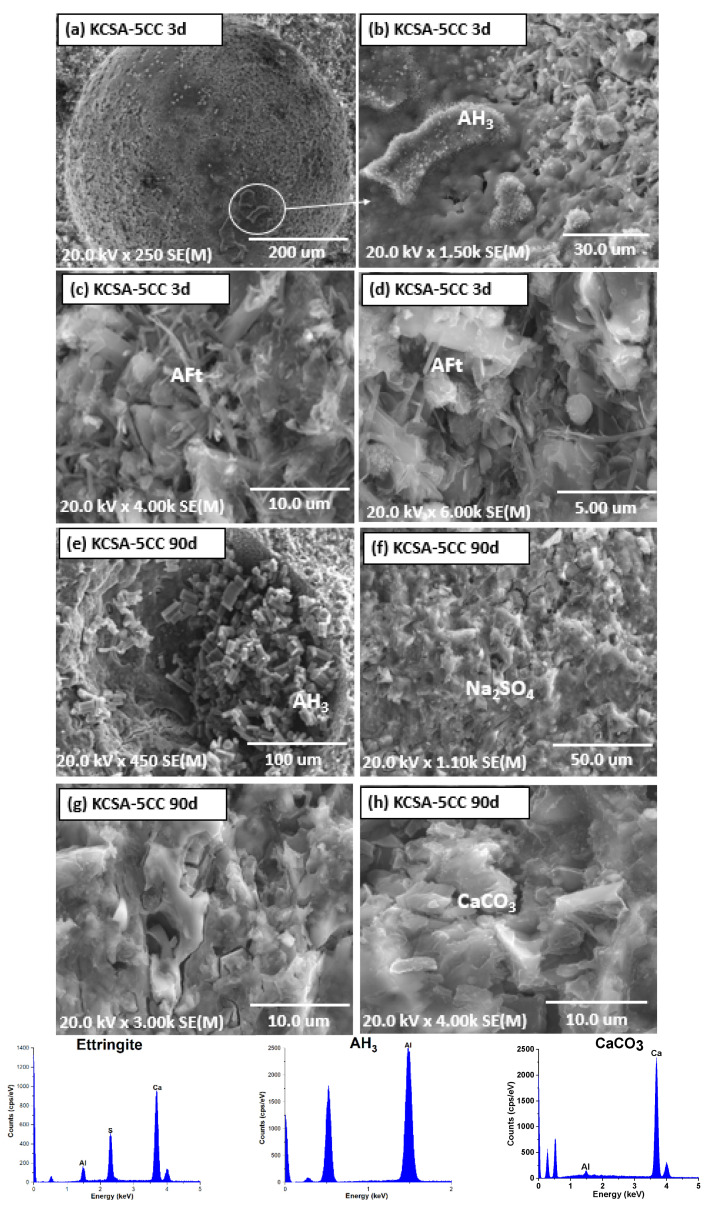
SEM microscopy of KCSA-5CC: (**a**–**d**) 3 days; (**e**–**h**) 90 days.

**Figure 9:**
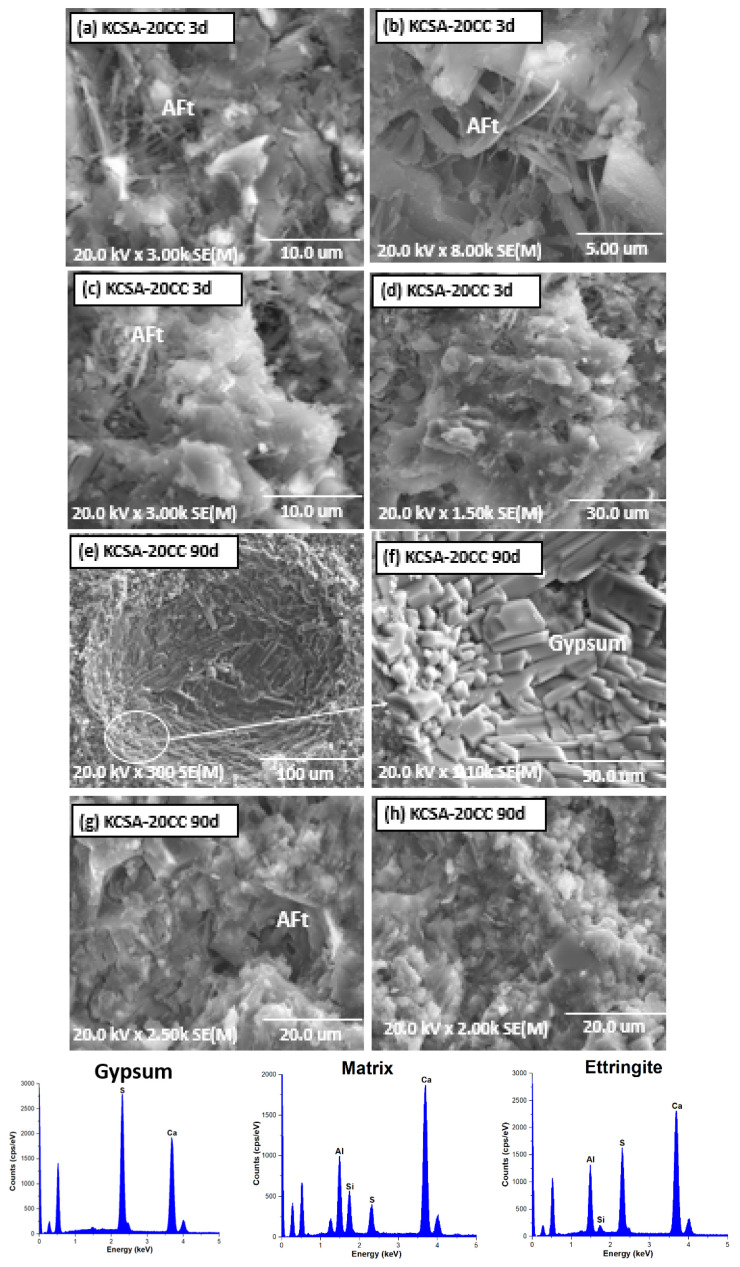
SEM microscopy of KCSA-20CC: (**a**–**d**) 3 days; (**e**–**h**) 90 days.

Figure 8 and Figure 9 show the micrographs of KCSA-5CC and KCSA-20CC pastes with 5% and 20% calcium carbonate at 3 days and 90 days respectively.

In the KCSA-5CC paste at 3 days, the formation of ettringite with a low degree of crystallisation is observed together with aluminium hydroxide and calcite plates (see Figure 8a–d). After 90 days, further precipitation of ettringite with a needle or rod morphology is observed, together with aluminium hydroxide and calcium carbonate (see Figure 8e–h). The formation of these phases contributes to the densification of the matrix after 90 days and increases the mechanical strength as shown in Figure 3.

In these pastes with calcium carbonate, an aluminium hydroxide with an amorphous to semi-crystalline morphology is observed. Other authors [35,48] indicate that an amorphous AH_3_ improves the mechanical properties. When the AH_3_ is of low crystallinity, it fills the spaces between the ettringite needles and improves the compaction of the matrix. In these pastes with 5 and 20% CaCO_3_, it is observed that both ettringite and aluminium hydroxide partially fill the voids, which would also indicate the continuous dissolution of the ye’elimite.

As the calcium carbonate concentration increases to 20%, the paste at 3 days (Figure 9a–d) shows denser matrices with the presence of ettringite needles. As the hydration increases to 90 days (Figure 9e–h), the formation of prismatic gypsum crystals in the pores is observed and ettringite is also detected.

As with aluminium hydroxide, the structure of ettringite varies in morphology depending on the hydration time and the percentage of calcium carbonate used. At 3 days in the KCSA-5CC paste, the formation of small volume ettringite needles is observed, which increase in size and frequency with increasing hydration time. As the salt concentration is increased, more voluminous needles are observed. This behaviour has been observed by other authors [10,49] who indicate that low CaCO_3_ concentrations result in a dense, spherical ettringite mixed with short, thin needles, and conversely, high CaCO_3_ concentrations result in the formation of ettringite with a long, fibrous needle morphology, which becomes shorter and thicker with increasing [Ca^2+^] ions in the starting solution.

The variation in ettringite morphology also explains the increase in mechanical strength at all ages studied in KCSA-5CC and KCSA-20CC, as well as the formation of a first peak before the induction period (1.4 h) in the heat release curve (Figure 1c), associated with the formation of a primary ettringite of low crystallinity. After the induction period, several peaks are formed associated with the precipitation of ettringite with a more defined morphology together with mainly aluminium hydroxide and other secondary hydration phases. These results are confirmed by XRD, FTIR and DTA characterisation.

The formation of gypsum in the calcium carbonate pastes may be due to an excess of calcium in the solution which, together with the sulphates from the ye’elemite, causes the precipitation of gypsum. However, considering that the added calcium carbonate is not very soluble and that the calcite content increases over time, a carbonation process may be occurring. Ettringite can react with atmospheric CO_2_ and decompose to produce gypsum and alumina gel (see Equation (1)) [50,51]:

3CaO · Al_2_O_3_ · 3CaSO_4_ · 32H_2_O + 3 CO_2_ (g) → 3 CaSO_4_ · 2H_2_O + 3 CaCO_3_ + 2 Al(OH)_3_ + 23 H_2_O (1)

**Figure 10:**
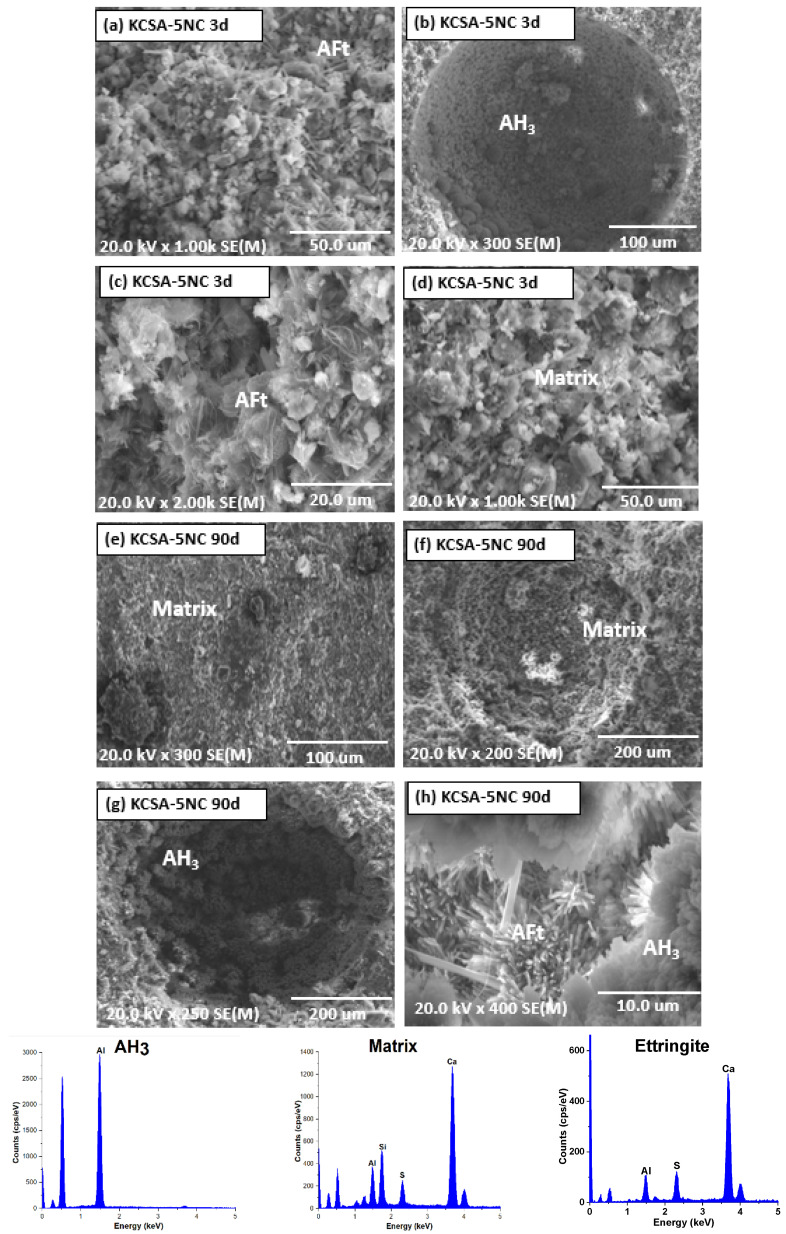
SEM microscopy of KCSA-5NC: (**a**–**d**) 3 days; (**e**–**h**) 90 days.

**Figure 11:**
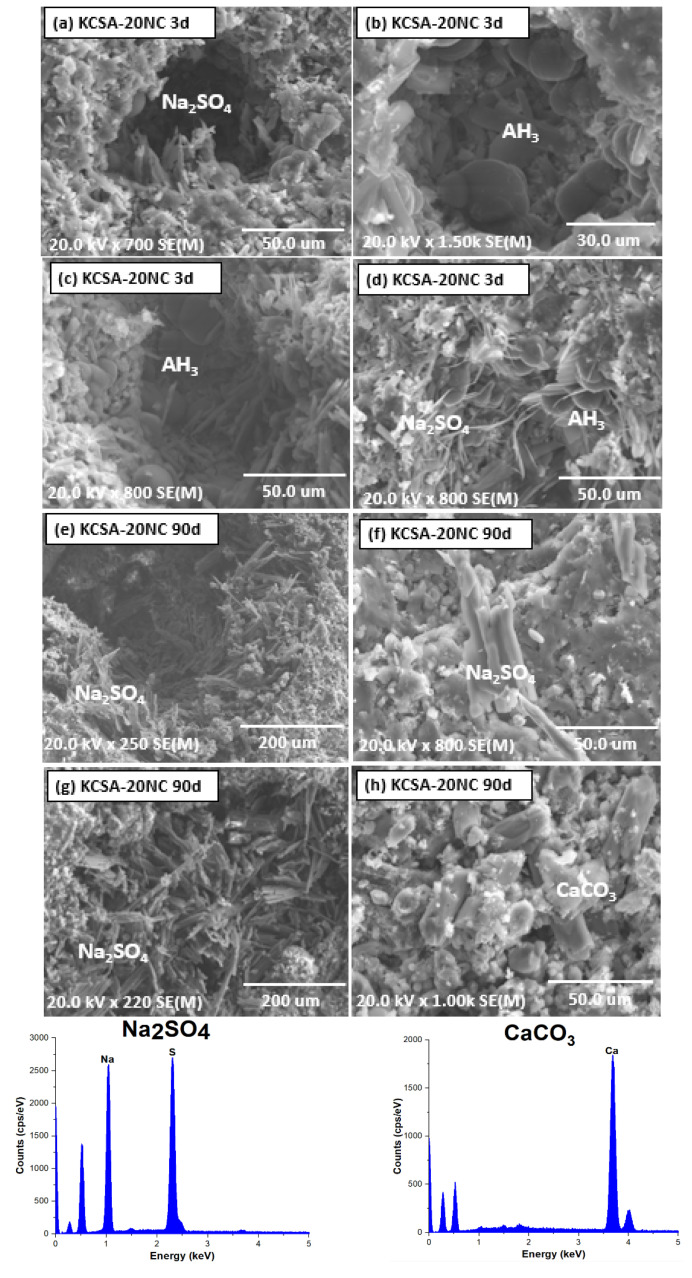
SEM microscopy of KCSA-20NC: (**a**–**d**) 3 days; (**e**–**h**) 90 days.

Figure 10 and Figure 11 show the micrographs associated with the 5% and 20% sodium carbonate pastes at 3 and 90 days of hydration. In the KCSA-5NC paste at 3 days (Figure 10a–d) the formation of poorly defined ettringite needles together with aluminium hydroxide in the form of spheres in the pores can be observed. With increasing hydration time (Figure 10e–h) the ettringite needles become more defined and elongated and precipitate together with aluminium hydroxide.

When the sodium carbonate concentration is increased to 20% (KCSA-20NC, Figure 11), the presence of larger prismatic particles corresponding to thenardite (Na_2_SO_4_) together with larger spheres associated with AH_3_ is observed in the micrographs (see Figure 11a–d) after 3 days of hydration (see Figure 11a–d). The presence of thenardite at 3 days was not detected by XRD, but was detected by microscopy. At 90 days, greater thenardite formation is observed by microscopy (Figure 11e–h), as well as plaques associated with calcium carbonate.

Sodium carbonate is highly soluble and can release Na^+^ ions in solution, raising the pH of the medium. An increase in pH delays the formation and precipitation of ettringite. This delay occurs in the KCSA-5NC sample. However, when the amount of Na_2_CO_3_ is high (KCSA-20NC), the amount of Na^+^ and carbonate ions entering the medium is much higher. The excess of sodium, together with the sulphates and calcium from the dissolution of ye’elimite, causes saturation of the medium and precipitation of gaylusite, CAH_10_ and thenardite. The presence of thenardite was not detected by XRD after 3 days, but by SEM. As the sodium ion content decreases due to the precipitation of gaylusite and thenardite, the precipitation of ettringite is favoured (signal detected at 28 days in the KCSA-20NC diffractograms). Both gaylusite and ettringite are not stable and evolve to form calcium carbonate. It is known [30] that ettringite can react with sodium carbonate to form thenardite, calcite and AH_3_ phases (see Equation (2)), which are clearly observed by XRD at 90 days. Thenardite leaches out as efflorescence (although it was clearly seen by microscopy). The presence of thenardite is associated with cracking and sample degradation [10,36].

(2) $$3\;\mathrm{CaO}\;\cdot \;{\mathrm{Al}}_{2}O_{3}\;\cdot \;3{\mathrm{CaSO}}_{4}\;\cdot \;32\;H_{2}O+6\;{\mathrm{Na}}^{+}+3\;{CO}_{3}^{2-}\to 3\;{\mathrm{CaCO}}_{3}\;+\;3\;{\mathrm{Na}}_{2}{\mathrm{SO}}_{4}\;+\;{\mathrm{Al}}_{2}O_{3}\;\cdot \;x\;H_{2}O\;+\;(32-x)\;H_{2}O$$

It is important to distinguish between the carbonation of ettringite by atmospheric CO_2_ (Equation (1)) and carbonation in the presence of sodium carbonate (Equation (2)), because although in both cases calcite and AH_3_ are formed, the precipitation of sulphates forms different compounds. In the first case the sulphates precipitate as gypsum and in the second case as thenardite. Therefore, in the KCSA-CC samples the strengths increase after 90 days, whereas in the KCSA-NC samples the strengths decrease after 90 days.

## 3. Materials and Methods

### 3.1. Materials

The i.tech ALI PRE GREEN industrial calcium sulfoaluminate clinker (KCSA) used in this study was supplied by HeidelbergCement Hispania (Madrid, Spain). The chemical composition was determined by X-ray fluorescence (40.82% CaO; 29.37% Al_2_O_3_; 9.92% SO_3_; 9.51% SiO_2_; 4.13% MgO, the rest corresponding to minority oxides such as Fe_2_O_3_, Na_2_O, TiO_2_, etc.). The loss on ignition of the KCSA clinker was 1.31%. The mineralogical composition, determined by XRD using the Rietveld method, was: ye’elimite = 68.4%; belite = 16.90%; bredigite = 7.40%, periclase = 3.67% and minority phases such as gehlenite and calcium aluminate. For further information see reference [10].

Particle size analysis of the calcium sulfoaluminate clinker was carried out by laser diffraction using a COULTER LS 130 particle size analyser with a measuring range between 0.1 and 900.0 µm. The results show that 90% of the particles are smaller than 35 µm. The density is 2.8318 g/mL and the Blaine fineness is 408.82 m^2^/kg.

### 3.2. Methods

Mixtures of calcium sulfoaluminate clinker (KCSA) were prepared with Panreac 99.8% reagent grade CaCO_3_ and Panreac 99.8% reagent grade Na_2_CO_3_. The proportions used were 5% or 20% by weight (95/5 and 80/20 mixtures). These mixtures are designated KCSA-5CC and KCSA-20CC when CaCO_3_ is used and KCSA-5NC and KCSA-20NC when Na_2_CO_3_ is used.

They were dry mixed in a shaker-mixer for one hour. These mixtures were hydrated with distilled water at a water/solid ratio of 0.5. The KCSA + salt + water mixtures were automatically stirred at 350 rpm for 3 min. Note that the KCSA paste hydrated with water only was used as the reference system (KCSA-H) in the mechanical strength and isothermal conduction calorimetry tests.

In order to determine the mechanical strength of these pastes, prismatic specimens of 1 × 1 × 6 cm^3^ were prepared [10]. For this purpose, a metal mould was used containing six specimens of different age and composition. Firstly, half of the mould is filled and compacted with 60 blows on the vibrating table, then a second layer is added and compacted with another 60 blows to homogeneously distribute the paste and prevent the formation of pores. The pastes are cured in a climatic chamber (24 h at 21 °C and 95% relative humidity). At the age of breaking (3, 28 and 90 days), the specimens are broken under pressure using an Ibertest press (Autotest-200/10-SW, Madrid, Spain).

Once the samples are crushed, part of the solid is ground in an agate mortar, mixed with isopropanol in a ratio of 1:10 (solid: isopropanol), the mixture is shaken for 3 min and filtered. It is then dried in a vacuum desiccator for 3 days. Some of the unground sample pieces are placed in isopropanol and left in solution for 7 days, then dried in a desiccator. The powder is used for X-ray diffraction (XRD), Fourier transform infrared spectroscopy (FTIR), thermal differential analysis (TDA/TG) and the unground pieces for scanning electron microscopy (SEM/EDX).

The XRD analysis was carried out using a BRUKER D8 ADVANCE (Madrid, Spain) model with a 3 KW high voltage generator, a copper anode X-ray tube (Cu Kα_1,2_ radiation of 1.540Å) normally operating at 40 Kv and 50 mA. The detector is a Lynxeye (Madrid, Spain) with a 3 mm antiscatter slit and a Ni K-beta filter (0.5%), without monochromator (does not remove Kα_2_). The qualitative analysis is carried out with a 6 mm variable divergence slit in the range 5–60° 2θ with a time/step of 0.5 s and a step size of 0.02°.

An ATI MATTSON GENESIS spectrophotometer was used for the FTIR analysis. Samples were prepared using 1.0 mg of test sample and 200 mg of KBr. The frequency range covered was 4000 to 400 cm^−1^. The infrared spectrum was acquired with 32 scans and a spectral resolution of 4 cm^−1^. The wavelength accuracy is 0.01 cm^−1^ for each data point.

DTA/TG analysis was used to determine the physicochemical changes of the materials with temperature and their weight variations. The instrument used was a TA SDT Q 600 (New Castle, DE, USA). The recording conditions were heating from room temperature to 1000 °C at 10 °C/min in a platinum crucible under a nitrogen atmosphere. The sensitivity of the measurement is 0.001 °C for DTA and 0.1 µg for TGA.

A HITACHI S-4800 field emission gun (FEG) microscope (Madrid, Spain) with a resolution of 1.4 nm was used for the SEM/EDX studies. This instrument has a backscattered electron detector (BSEM) (Madrid, Spain), a Bruker RX detector (Madrid, Spain), a microanalysis program (QUANTAX 400) and five monitored axes. The samples were dried under vacuum and metallised with carbon.

A study of the rate of heat release and the total heat associated with the hydration reactions was also carried out by isothermal conduction calorimetry using a THERMOMETRIC TAM AIR at 25 °C (Madrid, Spain). For this purpose, pastes with the same water/solid ratio of 0.5 were prepared. The reference used was distilled water.

## 4. Conclusions

In this work, the effect of the concentration and type of carbonate on the hydration of a commercial KCSA at 3, 28 and 90 days of hydration was studied. The general conclusions that can be drawn from this study are summarised below:

- The presence of CaCO_3_ does not significantly affect the hydration kinetics of KCSA, which behaves similarly to the reference with both 5% and 20% CaCO_3_, this behaviour being mainly related to a filler effect. The mechanical strength values increase progressively with hydration time and exceed those of the reference system after 90 days. The reaction products formed are those normally observed in the hydration of KCSA, ettringite, AH_3_ and, to a lesser extent, CAH_10_. However, a certain degree of carbonation of ettringite is observed over time, leading to the formation of calcite, gypsum and alumina gel, which densify the matrix and increase strength. These results suggest that the incorporation of CaCO_3_ (possibly as limestone) may have a beneficial effect on the development of the mechanical strength of KCSA.
- The presence of Na_2_CO_3_ has a negative effect on the hydration kinetics of KCSA, especially at low concentrations. The mechanical strengths are lower than those obtained with the reference. The amount of carbonate added also influences the reaction products formed. At low proportions, there is a long delay in the formation and precipitation of ettringite (similar to the behaviour observed in the hydration of KCSA with 1 M NaOH). The initial strengths are very low, but increase at 28 and 90 days due to the formation of ettringite and AH_3_. At longer ages, some carbonation is also observed to form calcite, thenardite and alumina gel.
- High levels of Na_2_CO_3_ (20%) initially inhibit the formation of ettringite and favour the formation of calcium aluminate, gaylusite and thenardite. When the sodium content is reduced, ettringite is formed (28 days), but as these are metastable reactions, the gaylusite and ettringite decompose to form calcite, alumina gel and a large amount of thenardite, which leaches out as efflorescence, causing microcracking and loss of strength in the material. These results indicate that the incorporation of Na_2_CO_3_ can have a detrimental effect on the mechanical strength development of KCSA.

## Data Availability

Data are contained within the article.
